# Involving patients, family, partners and/or carers in measuring and monitoring patient safety in hospitals: Protocol for an umbrella review

**DOI:** 10.12688/hrbopenres.14428.1

**Published:** 2026-04-30

**Authors:** Róisín O'Malley, Caoimhe Madden, Paul O'Connor, Warren Reilly, Elaine Toomey, Tom Reader, John Fitzsimons, Ronan Mahon, Sinéad Lydon

**Affiliations:** 1 Discipline of General Practice, School of Medicine, University of Galway, Galway, County Galway, Ireland; 2 Irish Centre for Applied Patient Safety and Simulation, School of Medicine, University of Galway, Galway, County Galway, Ireland; 3 Discipline of Medical Education, School of Medicine, University of Galway, Galway, County Galway, Ireland; 4 Centre for Health Research Methodology, School of Nursing and Midwifery, University of Galway, Galway, County Galway, Ireland; 5 Department of Psychological and Behavioural Science, The London School of Economics and Political Science, London, England, UK; 6 Children's Health Ireland, Temple Street, Dublin, Ireland; 7 Clinical Director for Quality Improvement, National Quality and Patient Safety, Health Service Executive, Dublin, County Dublin, Ireland; 8 Discipline of Economics, School of Business and Economics, University of Galway, Galway, County Galway, Ireland

**Keywords:** Patient Safety, Patient Participation, Patient Involvement, Family Participation, Safety Measurement, Umbrella review, Review of reviews

## Abstract

**Background:**

While substantive efforts have been made to improve the safety of hospital care, these have largely targeted the healthcare professional. Such a focus ignores the patient’s ‘voice’, and the unique insights patients and their families have into healthcare system functioning. Despite international policy shifts toward empowering patients to support patient safety, there remains a lack of knowledge regarding the full range of existing approaches for facilitating patient involvement in measuring and monitoring patient safety (MMS) in the hospital. This umbrella review will aim to identify existing approaches to involving adult inpatients (or family/partners/carers communicating on their behalf ) in MMS in hospital settings, along with evidence supporting their use.

**Methods:**

The current review will adhere to the Joanna Briggs Institute guidance for umbrella reviews and the Preferred Reporting Items for Overviews of Reviews (PRIOR) checklist. Systematic searches will be conducted across five electronic databases (MEDLINE, CINAHL, PsycINFO, Scopus, and Academic Search Complete) to identify existing reviews that examine patient-involvement in MMS in hospital settings. Data will be extracted on the characteristics of approaches identified (e.g., surveys, interviews, incident reporting) along with any evidence of their feasibility, contextual appropriateness and psychometric properties. Methodological quality will be appraised using the CASP checklist, and certainty of evidence will be assessed via a hybrid GRADE and GRADE-CERQual approach.

**Conclusions:**

This umbrella review will deliver a comprehensive profile of each different approach to involving patients, families and carers in MMS in hospital settings. By evaluating the psychometric evidence and practical feasibility (affordability, equity, and acceptability) of these approaches, the findings will support the ascertainment of best practice to involving patients in MMS in hospital settings, ultimately supporting an important shift in the framing of patients as passive recipients of care to active partners in safe and effective care delivery.

## Introduction

The patient ‘voice’, and the potential for patients to provide data to support patient safety improvement in hospitals, has largely been ignored. ^1, 2^ Current practices position the patient as passive, merely experiencing safety incidents and healthcare delivery, and fail to recognise the active role patients can play in ensuring safe care. ^3^ Patients and their families have substantial insight into healthcare system functioning, observing all healthcare interactions. ^2^ They have privileged access to information on continuity of care, communication failures, and respect issues. ^4^ Further, patients and families experience important elements of healthcare that are not observable to healthcare workers, such as admission processes and post-discharge care. ^4^ Patients can also provide preventative information on near misses or behaviours they perceive as concerning (e.g., poor infection control), and are well-placed to capture the nuanced harms associated with adverse events, including psychological and financial harm. ^2^ A variety of ways in which patients can contribute to improving patient safety have been identified. ^3^ One such mechanism is through participating in safety data collection.

Consistent data collection and analysis is required to identify areas where intervention is required to improve safety. ^5^ Despite the valuable insights that patients, their families and carers, may have in relation to the quality and safety of care delivered, they are not routinely provided with such opportunities to provide safety data. ^2^ To address this need, a range of methods have been developed that proactively gather data on patients’ perceptions of safety conditions, risks or hazards, or safety behaviours while in the hospital. For example, patients can complete surveys to assess the safety of their care, ^6^ bedside interviews to discuss safety concerns, ^7^ or review their clinical notes for errors. ^8^ There are also reactive methods which involve patients in gathering data following adverse events. These include the analysis of complaints and patient incident reporting tools. ^1, 7^ Where opportunities to provide safety data are available, research suggests that patient participation is high. ^9^

This importance of providing, and embedding, opportunities for patient involvement in safety measurement has been emphasised in a recent Lancet editorial. ^10^ ‘Engaging patients and families as partners in safety care’ has been cited as a guiding principle of the WHO’s Global Patient Safety Action Plan 2021–2030. ^11^ In the US, implementation of a patient reporting tool has been identified as a national goal. ^8^ In the UK, the National Patient Safety Agency includes patient reports of safety incidents in its National Reporting and Learning System. ^1^ In Ireland, the first strategic commitment within the Health Service Executive’s Patient Safety Strategy ^12^ is “Empowering and Engaging Patients to Improve Patient Safety”, and developing scalable mechanisms for patient input and formalising the role of families and carers in the safety process is urged. The importance of the patient’s ‘voice’ to supporting and improving patient safety has also been emphasised by many patient organisations and networks in the UK, ^13^ the US, ^14^ and internationally. ^15– 17^

Although the importance of involving patients in measuring and monitoring patient safety (MMS) is therefore well-recognised, there remains a lack of knowledge regarding the full range of approaches that exist for facilitating patient involvement in MMS, as well as the specific contextual factors, including practicability and equitability, that should guide their selection. ^1, 18, 19^ Understanding the full range of approaches for involving patients in MMS is essential as patients’ willingness to provide safety data, ^20^ and the barriers they encounter when providing safety data ^20– 22^ differ according to the data collection method used. Previous reviews have examined approaches to involving patients in MMS. For example, Madden et al. ^23^ and Li et al. ^19^ explored the psychometric properties and validity of patient-reported safety climate and participation questionnaires. Others have narrowed their scope to the reporting of adverse events, either by examining the means for eliciting these incidents ^1^ or by reviewing the technical reporting systems used to solicit those reports. ^24– 26^ Furthermore, Ramsey et al. ^27^ explored the qualitative perspectives of stakeholders regarding patient involvement in serious incident investigations. Despite these important contributions, the existing literature on involving patients in MMS remains limited by a focus on individual methods in isolation, leaving the full spectrum of patient safety data collection currently unexamined. Truly comprehensive MMS must be holistic and multidimensional, incorporating retrospective learning from past incidents with real-time data on the reliability of processes, the sensitivity to operations, and the capacity for organisational learning. ^5, 28^ Accordingly, considering the range of existing approaches, and the characteristics of, and evidence supporting, each approach is crucial for furthering the ability to involve patients in MMS on-the-ground.

## Objectives

This review will employ an umbrella review methodology ^29, 30^ in order to identify, and develop an understanding of, existing approaches to involving patients in MMS that have been used within adult inpatient hospital care. Umbrella reviews allow for the integration of evidence from multiple systematic reviews to provide a comprehensive overview of a broad research area where several prior syntheses already exist. ^29, 30^ While a number of reviews in recent years have considered different means of involving patients in safety data collection, ^1, 19, 23– 27^ synthesising the full literature describing the various different approaches to involving patients in MMS will support an understanding of the extent of different approaches, their characteristics, and what might be recommended as current best practice for involving patients in MMS in hospital settings. Accordingly, our review question is as follows:

What approaches to involving adult inpatients (or family/partners/carers communicating on their behalf ) in measuring and monitoring patient safety in hospitals have been used to-date, and what is the evidence supporting their use?

The primary review objectives will be to:

1. Identify, and profile, all approaches that have been used to involve patients (or alternatively family, partners, or caregivers on their behalf ) in MMS in hospital-based care,
2. Collate any data presented on the Affordability, Practicability, Effectiveness/cost-effectiveness, Acceptability, Side-effects, and Equity ^31^ of approaches identified, and
3. Summarise any data presented on the psychometric evidence of existing approaches and tools.

The development of this review question has been supported by application of the FINER criteria ^32^ to confirm it is appropriately feasible, interesting, novel, and relevant and will comprise a valuable addition to the research literature and a useful support for practice.

## Methods

### Review design and protocol registration

Umbrella reviews (also commonly referred to as overview of reviews, review of reviews, meta-reviews ^30, 33^) serve to gather, assess, and synthesise evidence from multiple reviews on a specific topic, ^30^ in order to aggregate evidence across multiple syntheses, identify patterns in the results, and evaluate whether the existing reviews offer consistent or conflicting evidence. ^29^ This is important as multiple existing reviews on a given topic complicate evidence-based decision making and are challenging for end users to contend with. ^30, 33, 34^ Consequently, an umbrella review methodology was adopted, given their suitability for topics that have been researched or reviewed previously and for which the determination of evidence-based, or best, practice is important. Existing evidence syntheses in the area ^1, 19, 23– 26^ have been limited by a focus on individual methods and single dimensions of safety in isolation, ^5^ and an umbrella review will comprise an importance support for progressing efforts to involve patients in MMS.

This umbrella review will be conducted in line with the Joanna Briggs Institute (JBI) methodological guide for umbrella reviews ^35^ and best practice guidance for the conduct of umbrella reviews. ^33– 35^ Further, the review will be reported in accordance with the Preferred Reporting Items for Overviews of Reviews (PRIOR) checklist. ^30^ The current protocol was prepared as per the Preferred Reporting Items for Systematic Reviews and Meta-Analysis Protocols (PRISMA-P) statement, ^36^ coupled with careful attention to the JBI Reviewer’s Manual ^35^ and PRIOR checklist. ^30^ The PRISMA-P checklist has been provided in *Extended data,* Appendix 1. The protocol was registered with the International Prospective Register of Systematic Reviews, PROSPERO.

### Search strategy

Searches will be conducted across five electronic databases, specifically: MEDLINE (OVID); CINAHL (EBSCO); PsycINFO (EBSCO); Scopus (Elsevier), and; Academic Search Complete (EBSCO). The search strategy was developed by the research team, and includes Subject Headings as well as free-text keywords related to: 1) patient safety (e.g., exp Patient Safety/, ‘patient safety’, ‘patient harm’), 2) patient, family, partner and carer involvement (e.g., exp Patient Participation/, ‘patient participation’, ‘family involvement’), and 3) the use of a review methodology (e.g., ‘systematic review’, ‘meta-analysis’). A search strategy was initially developed for MEDLINE (see Table 1 for MEDLINE search strategy) and adapted as necessary for use in the other databases (see *Extended data,* Appendix 2 for search strategies used across all electronic databases). The development of the search strategy was informed by consideration of previous reviews of patient-report patient safety measurement tools that were known to the review team, ^1, 2, 19, 23– 26^ as well as umbrella reviews of measurement tools for use in healthcare. ^37– 39^ In addition, the search strategy has been reviewed and agreed by a University research librarian. Consideration of PRESS (Peer Review of Electronic Search Strategies ^40^) and reporting standards for literature searches by Atkinson and colleagues’ ^41^ has supported us in ensuring search quality, transparency and replicability.

**Table 1.** Search strategy for Medline (OVID) database.

**1.**	exp Patient Safety/
**2.**	exp Patient Harm/
**3.**	(patient* adj1 (harm or safe*2)).ti,ab.
**4.**	exp Medical Errors/
**5.**	((medic* or human or health?care) adj1 error*1).ti,ab.
**6.**	(preventable or avoidable or unintended or unnecessary) adj1 (harm or consequence*1).ti,ab.
**7.**	(safe*2 adj1 (care or culture or climate or opportunit*)).ti,ab.
**8.**	exp Iatrogenic Disease/
**9.**	(iatrogenic adj1 (harm or disease)).ti,ab.
**10.**	**1–9/OR**
**11.**	Exp Systematic Review/
**12.**	Exp Scoping Review/
**13.**	Exp Meta-Analysis/
**14.**	((systemati* or scoping or rapid or literature) adj1 review).ti,ab.
**15.**	(meta?analys*).ti,ab.
**16.**	(meta adj1 analys*).ti,ab.
**17.**	((search* or synthes*) adj3 (literature or research or evidence or database*1)).ti,ab.
**18.**	**11–17/OR**
**19.**	exp Patient Participation/
**20.**	exp Patient Reported Outcome Measures/
**21.**	((patient*2 or family or families or carer*1 or partner*1) adj2 (participation or perception* or perceive* or report* or involv* or engag* or concern* or complain* or comment* or review* measure* or monitor* or feedback or input* or survey* or questionnaire* or knowledge or insight*1 or interview*2 or experience*1 or access*)).ti,ab.
**22.**	**19–21/OR**
**23.**	**10 AND 18 AND 22**

To complement electronic database searches, both backward and forward citation searching, as per best practice recommendations, ^42^ will be employed. First, the reference lists of all studies deemed eligible for inclusion following the initial electronic searches will be screened. Similarly, the reference lists of a previous umbrella review ^43^ and systematic reviews ^44– 47^ that explored patient engagement in healthcare safety more broadly will also be reviewed to identify any additional relevant review papers. Second, using the included studies and the aforementioned reviews, ^43– 47^ we will conduct forward citation searching using Scopus and Google Scholar. These two citation indexes were selected to ensure a comprehensive capture of more recent work. All records identified through these methods will be tracked and reported separately in the PRISMA flow diagram. ^42^

### Eligibility criteria

Eligibility criteria are outlined according to the PICo-T format for umbrella reviews, ^35^ comprising the Population (P), Phenomena of Interest (I), Context (Co), and Types of Studies (T). Accordingly, our eligibility criteria are as follows:

***Population.*** The population of interest, within relevant reviews, will include adults (defined as individuals aged over 18 years) receiving care within an inpatient hospital setting. Recognising the valuable insight of the patient’s family and carers, we will also include reviews that engage family members, partners, or carers in the patient’s stead. Conversely, we will exclude reviews that focus solely on the MMS safety through engagement with other stakeholders, such as healthcare providers, without direct patient, family or carer involvement. Further, while some reviews may span multiple healthcare settings (e.g., both paediatric and adult populations), we will exclude those where data specific to adult inpatients cannot be clearly extracted.

***Phenomena of Interest***. The phenomenon of interest is the involvement of patients (or family members, partners, or carers) in MMS in inpatient hospital care settings. For reviews which describe a broader focus on patient involvement in patient safety (e.g., interventions designed to enhance safety through patient participation, of which involving patients in MMS is just one potential form/element), two independent reviewers will systematically consider the full-text manuscript to determine whether a clear focus within the study (e.g., within the study aim or data extraction) relates to patient involvement in MMS, and whether relevant data on such approaches are included and can be extracted. Conversely, we will exclude reviews that do not specifically synthesise approaches to MMS from the patient, family, partner or carer perspective. We will also exclude reviews where the focus of the approaches, tools, or instruments are broader than patient safety (e.g., general quality of care or patient experience), unless they contain at least one patient safety-specific subscale or section for which data can be extracted. Finally, we will exclude reviews of studies that rely upon soliciting patient input in response to hypothetical vignettes and scenarios only, or those designed to capture general opinions on patient safety rather than focusing on specific patient experiences, and the elicitation of these, related to safety.

***Context***. This umbrella review is specifically focused on patient safety within the context of adult inpatient hospital care. We define patient safety in accordance with the World Health Organisation ^48^ as “the prevention of errors and adverse effects to patients associated with health care.” Inpatient hospital care is characterised by the formal admission of a patient to a healthcare facility for treatment, diagnostic tests, or surgical procedures requiring a stay of at least one night (typically 24 hours) that concludes with a formal discharge, occurring when the patient returns home, is transferred to another facility, or in the event of death. ^49, 50^ While we recognise the importance of safety across the continuum of care, other healthcare domains, including primary and community care, outpatient services, and paediatric or maternity care, differ significantly in their patient population, and clinical and operational structures. Consequently, research relating to these settings, falls outside the scope of this review.

***Study Type***. Systematic reviews, and comprehensive reviews of any design type (e.g., scoping reviews, rapid reviews) with evidence of a systematic search strategy in at least two databases will be included, similar to previous mixed-method umbrella reviews of healthcare research. ^51– 53^ Narrative review articles that do not follow a systematic approach, and primary studies will be excluded. There will be no restriction by country or publication year, though publications are required to be peer-reviewed and written in the English language.

### Study selection

Studies retrieved in electronic database searches will be first exported into Endnote© ( https://end-note.com/; Zotero ( https://www.zotero.org/) may be considered as a free alternative that can perform equivalent functions) to support the removal of duplicate studies as per Bramer et al.’s de-duplication process. ^54^ Subsequently, all remaining records will be exported to Microsoft Excel© ( https://www.microsoft.com/en-ie/microsoft-365/excel; Google Sheets ( https://www.google.com/sheets/about/) may be considered as a free alternative that can perform equivalent functions) and screened according to their titles and abstracts. ROM and SL will first pilot the eligibility criteria on a random sample of the titles and abstracts of 10 reviews, similar to Moens et al. ^38^ Subsequently, ROM will screen the remaining returns, whereby the full texts of articles judged to meet the inclusion criteria, or where there was insufficient information to determine eligibility, will be retained for full-text review. These will be screened by ROM and a final decision on inclusion will be made. During the full-text screening stage, the specific reasons for the exclusion of any studies will be systematically documented within Microsoft Excel© and summarised in the final PRISMA flow diagram ^55^ to ensure methodological transparency and rigor. If there is ambiguity surrounding the suitability of an article, this will be discussed by the two authors (ROM and SL), in consultation with the wider research team where necessary, until consensus is achieved.

### Data extraction

A data extraction form, informed by previous umbrella reviews ^38, 39, 43^ and systematic reviews of patient involvement in patient safety ^1, 19, 23^ has been developed. Table 2 presents the full range of variables for which data will be extracted. The form will be piloted in advance of beginning formal data extraction by two authors (ROM and SL). Where data are missing, this will be reported in the final review. Duplicate data extraction will be independently completed by two authors for each study, with disagreements resolved by discussion until consensus is achieved.

**Table 2.** Data extracted from the included review studies and method of synthesis.

Variables	Descriptions	Coding
**Data on the review’s methodology and findings**		
**Author(s)**	Last names of the author(s) of the review	N/A
**Publication year**	The year in which the review was published	N/A
**Review aim(s)**	The research questions, aims, and/or objectives of the review	N/A
**Period covered**	The publication period covered by the included primary studies	N/A
**Number of included studies**	The number of primary studies included in the review	N/A
**Quality assessment**	The assessment tool used by the author(s) to evaluate the quality of the included studies, and the score and interpretation of the quality of the included studies	N/A
**Data on approach/tool to involving patients in measuring patient safety**		
**Name of tool/approach**	The name (if any) of the approach (es)/tool(s) discussed in the review	N/A
**Type of safety measure**	The type of approach (es)/tool(s) used to collect data from patients	Classified through a deductive-inductive approach, ^78, 79^ with coding informed by existing frameworks, ^39, 80, 81^ while also allowing for novel types of approaches/methods to emerge de novo from the data.
**Description**	A short description of the approach (es)/tool(s) as reported by the author(s)	N/A
**Mode of delivery**	The medium or format through which the tool is administered (e.g., paper-based survey, face-to-face interview)	N/A
**Administration and frequency of measurement**	When and how often the measurement was administered	N/A
**Dimension of safety and type of safety issue**	The specific nature of the safety incident or risk the tool is designed to identify, monitor, or mitigate	Dimension of safety classified into past harm, reliability of safety critical processes, sensitivity to operations, anticipation and preparedness and integration and learning, according to the Measuring and Monitoring Safety (MMS) framework ^3, 28^ Type of safety issue classified through inductive coding
**Development of approach (es)/tool(s)**	The origin of the tool, including whether it was newly created for the study, adapted from an existing framework, or used in its original validated form. Detail on the design process, theoretical underpinning, or pilot testing used by the authors.	N/A
**Participants**	The participants who used the approach/tool to provide safety data	N/A
**Administrators/staff**	The people responsible for administering the approach/tool to the participants	N/A
**Setting**	The setting in which the tool was used	N/A
**Data on the validity and feasibility of the approach (es)/tool(s)**		
**Evidence on the feasibility and appropriateness of approach/tool**	All information reported on the Affordability, Practicability, Effectiveness/cost-effectiveness, Acceptability, Side-effects, and Equity of approaches	Classified into Affordability, Practicability, Effectiveness/cost-effectiveness, Acceptability, Side-effects, and Equity, according to the APEASE framework ^31^
**Psychometric properties of approach/tool**	The framework used by the author(s) to organise psychometrics-related evidence for the approach/tool and/or the approach/tool’s psychometric properties that were reviewed (only relevant if the authors categorised the psychometric evidence) All the information reported regarding the tool’s psychometric properties.	Classified into the following categories: validity, reliability, and usability, as per Moens et al. ^38, 83^

### Methodological quality assessment

The methodological quality of the included reviews will be critically appraised using the Critical Appraisal Skills Programme (CASP) systematic review checklist. ^56^ The CASP systematic review checklist focuses on the methodological validity and practical relevance of a review. ^56^ The CASP checklist was selected for its explicit guidance and methodological flexibility ^57– 59^ and ability to accommodate various review types, such as rapid and scoping reviews, without compromising on rigor. ^56^ The CASP checklist has been successfully applied in previous umbrella reviews of measurement tools. ^38, 39^ The CASP checklist has three parts: part A, are the results of the study valid? (five items); part B, what are the results? (two items); and part C, will the results help locally? (three items). Similar to previous umbrella reviews of measurement tools, ^38, 39^ two items will not be applied in our appraisal as they are specifically relevant to interventions: (7) “How precise are the results?”; and (8) “Can the results be applied to the local population?”. CASP items require a response of ‘yes’ (1 point), ‘no’ (0 points), or ‘can’t tell’ (0 points), returning a total score out of 7. To ensure accuracy, two reviewers will complete the quality appraisals for each review study in tandem, and any disagreements will be resolved through discussion or consultation with a third reviewer.

### Certainty of evidence

To assess the certainty of evidence, we will employ a dual-framework approach, applying GRADE ^60, 61^ to quantitative outcomes and GRADE-CERQual ^62, 63^ to qualitative findings. While some have advised against appraising the certainty of the evidence in mixed methods reviews, ^64, 65^ organisations such as the National Institute for Health and Care Excellence support the complementary use of GRADE and GRADE-CERQual ^66^ frameworks, and these frameworks have been applied in previous healthcare systematic and umbrella reviews. ^67– 73^ Applying both frameworks will provide a more robust and holistic evidence base for decision-making than a single-framework assessment could achieve. ^74^

This assessment will be conducted at the level of each distinct approach to measurement (e.g., surveys, interviews, incident reporting) to determine the robustness of each method identified. Following established methodological guidance, ^61, 63^ each approach will begin with a ranking of High certainty and is downgraded to Moderate, Low, or Very Low based on serious concerns across standardised domains. For quantitative data, downgrading will be based on methodological quality (informed by CASP scores), inconsistency of results across reviews, indirectness of the evidence, imprecision in findings, and potential publication bias. ^60, 61^ For qualitative data, confidence will be evaluated based on methodological limitations, the coherence of findings, the adequacy of the data, and the contextual relevance. ^63^ Two reviewers (ROM and SL) will perform these assessments in tandem, with discrepancies resolved through discussion (as is recommended when using the GRADE-CERQual ^63^) and consultation with a third reviewer where necessary. The final certainty ratings for each approach will be summarised in Summary of Findings tables ^60– 63^ to facilitate evidence-based decision-making. If both quantitative and qualitative data are provided for a specific data variable (e.g., qualitative and quantitative data for effectiveness/cost-effectiveness), a convergent-segregated approach will be conducted by performing separate quantitative and qualitative syntheses, then integrating the results derived from each of the syntheses. ^66, 75^

### Data synthesis

Given the anticipated diversity within the review papers included, content analysis ^76^ will be undertaken to synthesise the extracted data. This involves the development of categories/codes either de novo or through engagement with existing theories that are applied through the consideration of data extracted from each distinct study. ^77^ It is anticipated that content analysis will be used as part of data extraction and synthesis. This will comprise an initial stage of data synthesis, and will allow us to bring consistency of language across studies and extracted data and facilitate later comparison of studies. As presented in Table 2, it is anticipated that data pertaining to the following variables will be coded deductively by two authors independently during this process:

***Methods of measurement.*** Approaches to MMS from the perspective of patients, family members, and carers will be categorised using a hybrid deductive-inductive approach. ^78, 79^ While initial categorisation is informed by established frameworks (e.g., surveys, interviews, incident reporting, and record reviews) ^39, 80, 81^; this review recognises that these frameworks are largely derived from healthcare provider perspectives. Consequently, the classification will remain flexible and iterative, allowing for the identification and inclusion of novel, patient-led measurement approaches (e.g., narrative storytelling, real-time digital feedback) that may not be captured by traditional, healthcare professional-focused taxonomies.

***Dimension of safety.*** The dimension of patient safety measured by the study’s approach to safety measurement will be coded according to the five dimensions of the MMS framework, ^5, 28^ which includes past harm, reliability of safety critical processes, sensitivity to operations, anticipation and preparedness, and integration and learning. This framework is well-established in safety research as it allows for a multidimensional understanding of safety, which moves beyond retrospective incident reporting toward a more holistic, proactive view of safety system performance. ^5, 28^ Consequently, it is commonly used in reviews examining the MMS in healthcare. ^39, 80, 81^

***Feasibility and contextual appropriateness.*** Data provided on the feasibility and contextual appropriateness of the approaches to measurement will be coded according to the APEASE (Affordability, Practicability, Effectiveness/cost-effectiveness, Acceptability, Side Effects/Safety, Equity) framework. ^31^ This provides a structured lens to evaluate not just if a tool works in a vacuum, but if it is viable for use by patients, and their families, within the complex environment of an acute hospital. ^31^ When evaluating methods of MMS, the APEASE criteria provide a methodical way of assessing the acceptability, feasibility and contextual appropriateness of such approaches, ^31^ in that, if all six criteria are achieved then an approach or intervention is more likely to be successful and lead to behaviour change (i.e., patients will use the approach to provide safety data). ^82^

***Psychometric evidence.*** Evidence reported regarding the approach’s/tool’s psychometric properties will be categorised as validity, reliability, or usability, similar to the approach undertaken in Moens et al. ^38, 83^ Recognising that included reviews may vary significantly in their depth of reporting, we will not apply a rigid, granular psychometric threshold (e.g., as used in specialised tool-validation reviews like COSMIN ^84^). Instead, we will adopt a flexible approach that accounts for the anticipated diverse nature of identified methods. Many approaches to involving patients in MMS, such as patient-report surveys (often targeting past harm or anticipation and preparedness dimensions of the MMS framework ^5, 28^) may provide substantive psychometric data (e.g., the reviews by Madden et al. ^23^ and Li et al. ^19^). Conversely, emerging measurement approaches, particularly those aimed at assessing the ‘sensitivity to operations’ or ‘integration and learning’ dimensions of the MMS framework (e.g., safety walk-rounds or the integration of safety lessons into practice ^5, 28^), may prioritise practical utility and contextual responsiveness over formal psychometric properties. ^1^ Anticipating this variation, our synthesis will capture the breadth of evidence available, ensuring we do not inadvertently emphasise formal validation at the expense of capturing innovative, patient-informed approaches that are essential for holistic safety monitoring. ^3^

Following this, a narrative synthesis approach will be undertaken to analyse the resulting data, guided by Popay et al.’s ^85^ approach to narrative synthesis. A meta-analysis will not be performed as the focus of umbrella reviews remains on summarizing existing evidence syntheses rather than generating new pooled estimates. ^35^ Accordingly, different methods and tools will be used as part of this narrative synthesis, such as text-based summaries of studies, the categorisation of similar studies, the use of tables and figures to present summary data, and the transformation of data using content analysis. ^85^ We will assess and report the overlap of primary research studies across the included reviews to ensure transparency regarding the evidence base. ^30^

### Patient and Public Involvement (PPI)

This umbrella review is part of a wider programme of research (the Patient Involvement in Patient Safety (PIPS) project), the development of which was supported by the Irish Centre for Applied Patient Safety and Simulation’s (ICAPSS; https://www.universityofgalway.ie/icapss/) established PPI group. The research team, and protocol authorship, includes a patient representative (WR) who has contributed to protocol development and will support completion of the review. Further, PPI members will contribute to the development of accessible, lay-friendly summaries of the review’s findings, ensuring the findings can engage members of the public.

## Discussion

***Summary***. This umbrella review aims to identify all existing approaches to involving adult inpatients (or family/partners/carers communicating on their behalf ) in MMS in hospital settings, along with elucidating evidence supporting their use. Data will be synthesised as to understand the different approaches that exist to measuring patient safety from the patient’s perspective (e.g., surveys, incident reporting, or novel patient-led approaches) and the dimensions of safety targeted within these, along with any available evidence pertaining to the feasibility, contextual appropriateness and psychometric properties of existing approaches. The findings will help to elucidate the full range of patient-informed safety measurement approaches, along with important considerations surrounding their use. This will inform what is recommended as best practice for involving patients in MMS in hospital settings. Ultimately, this work seeks to realise the patient desire to contribute to their own safety, support the routine integration of patient insights into hospital systems, and uncover actionable insights that enhance care quality for patients and inform wider system improvements.

***Limitations***. As an umbrella review, the quality of our findings will be inherently dependent on the depth and rigor of the included reviews. Any omissions or data extraction errors in the original reviews will be reflected in this synthesis. Furthermore, by restricting our scope to existing reviews, we may overlook primary studies published very recently that have not yet been captured in a systematic synthesis. Additionally, while we focus on adult inpatient care, many existing reviews aggregate data across diverse healthcare settings (e.g., primary care, paediatrics, and maternity). The challenge of de-aggregating these data may limit the depth of setting-specific insights we can provide for the hospital setting. There is significant variability in how patient-involved safety measurement is defined, ranging from real-time bedside interviews to retrospective surveys. This heterogeneity, combined with the diverse psychometric properties of the tools, may make direct comparisons or the synthesis of data challenging. The exclusion of non-English language reviews may introduce a degree of publication bias. These factors will be considered when interpreting the findings of this review.

***Implications.*** Implications for best practices in hospital-based safety monitoring, healthcare policy, and future research, such as the need for more robust psychometric and feasibility testing, will be presented. Any limitations of the reviews included, such as a surface-level consideration of included studies, or a failure to consider important aspects of approaches such as APEASE variables or usability, as well as limitations of the umbrella review itself, will be transparently outlined. We anticipate that these findings will be used to inform international recommendations for best practices in patient-involved safety measurement and to guide the development, and testing, of more effective, real-world monitoring systems in adult inpatient settings.

## Dissemination

The findings of this umbrella review will be disseminated through the publication of a peer-reviewed manuscript. Additionally, findings will be presented via conference presentation(s) both nationally and internationally. We will also ensure, with the support of our PPI contributors, that the findings are disseminated appropriately to engender public engagement also.

## Data Availability

No underlying data are associated with this article. Zenodo: ‘Involving patients, family, partners and/or carers in measuring and monitoring patient safety in hospitals: Protocol for an umbrella review.’ https://doi.org/10.5281/zenodo.18959173 This project contains the following extended data: **Appendix 1.** PRISMA-P checklist **Appendix 2.** Full search strategy for all five electronic databases Data are available under the terms of the Creative Commons Attribution 4.0 International license (CC-BY 4.0). ^86^
