# Supplementary material for: Involving adult inpatients, family, partners and/or carers in measuring and monitoring patient safety in acute care hospitals: Protocol for an umbrella review
Source: HRB Open Res. 2026 Jul 25;9:45. Originally published 2026 Apr 30. [Version 2] doi: 10.12688/hrbopenres.14428.2 (PMC13560569; doi:10.12688/hrbopenres.14428.2)
Supplement: Peer review history

# Reviewer report: Reviewer response for version 2

**DOI:** 10.21956/hrbopenres.16002.r56789

**Published:** 2026-08-12

**Authors:** Leila Keshtkar

**Affiliations:** 1 University of Leicester, Leicester, England, UK

No further comments. Author addressed the comments.

Is the study design appropriate for the research question?

Yes

Is the rationale for, and objectives of, the study clearly described?

Yes

Are sufficient details of the methods provided to allow replication by others?

Yes

Are the datasets clearly presented in a useable and accessible format?

Partly

Reviewer Expertise:

Patient safety, communication, healthcare inequality, evidence synthesis, health services research.

I confirm that I have read this submission and believe that I have an appropriate level of expertise to confirm that it is of an acceptable scientific standard.

# Reviewer report: Reviewer response for version 2

**DOI:** 10.21956/hrbopenres.16002.r56788

**Published:** 2026-07-28

**Authors:** Ramkrishna Mondal

**Affiliations:** 1 All India Institute of Medical Sciences, Patna, bihar, India

The revised version of the current study protocol almost entirely fulfil all the recommendations made by the reviewers. It is now more scientifically valid and relevant to the topic. 

 By defining the study population as adult inpatients, family, partners and/or carers in acute care, it is now easy to identify the exact study population. 

 The title has revised including study population and the method to be used.

 The revised introduction section is now more preciously raises the research questions with three identified objectives and the relevant background information.

 The detail methodology highlighting eligibility criteria, search strategy, data extraction, coding, synthesis and analysis methods enlighten this section than previous one.

 Inclusion of possible outcome, limitations, barriers and facilitators, and impacts enriched the study protocol.

 Overall, the revised study protocol may be accepted with minor English language editing in few places.

Is the study design appropriate for the research question?

Partly

Is the rationale for, and objectives of, the study clearly described?

Yes

Are sufficient details of the methods provided to allow replication by others?

Partly

Are the datasets clearly presented in a useable and accessible format?

Partly

Reviewer Expertise:

Hospital Administration, Patient safety, Health technology

I confirm that I have read this submission and believe that I have an appropriate level of expertise to confirm that it is of an acceptable scientific standard.

# Reviewer report: Reviewer response for version 1

**DOI:** 10.21956/hrbopenres.15897.r55736

**Published:** 2026-06-05

**Authors:** Ramkrishna Mondal

**Affiliations:** 1 All India Institute of Medical Sciences, Patna, bihar, India

The study protocol described here, is an important and vital topic of patient safety. The rationale and the objective were narrated with relevant citations. Following few suggestions may be considered as below to improve the study, -

 1. The title should specify what is mean by 'patient safety in hospitals' i.e. there should be a clear boundary of the study population. Whether pre & post hospital care included or not, though protocol specify adult over 18 years with no upper limit, whereas, vulnerable group have more risk,  

 2. Based on the three main objective, first one is of identity and demographic profile in MMS in hospital based care, needs clarity as stated above. Second objective based on APEASE approach, but more details on methodology of each variable needs to be described properly. Third objective specifically based on psychometric evidence only, but what method used not clearly mentioned.

 3. The methods section need more explanation on how the data collected as stated above and what method used to analyse need to mentioned.

 4. A short note on expected outcome may be added.

Is the study design appropriate for the research question?

Partly

Is the rationale for, and objectives of, the study clearly described?

Yes

Are sufficient details of the methods provided to allow replication by others?

Partly

Are the datasets clearly presented in a useable and accessible format?

Partly

Reviewer Expertise:

Hospital Administration, Patient safety, Health technology

I confirm that I have read this submission and believe that I have an appropriate level of expertise to confirm that it is of an acceptable scientific standard, however I have significant reservations, as outlined above.

# Response

**Published:** 2026-07-14

**Authors:** Róisín O'Malley

**Affiliations:** Discipline of General Practice, University of Galway, Galway, Co. Galway, Ireland

**The study protocol described here, is an important and vital topic of patient safety. The rationale and the objective were narrated with relevant citations. Following few suggestions may be considered as below to improve the study, -**

 Thank you for your constructive review of our umbrella review protocol. We have found your suggestions and comments helpful and have provided a point-by-point response to your feedback below.

**1. The title should specify what is mean by 'patient safety in hospitals' i.e. there should be a clear boundary of the study population. Whether pre & post hospital care included or not, though protocol specify adult over 18 years with no upper limit, whereas, vulnerable group have more risk,**

**Answer: **The authors have revised the Title to clearly specify the study population as "adult inpatients... in acute care hospital settings," thereby explicitly excluding pre- and post-hospital care. We hope that this is satisfactory to the reviewer.

**2. Based on the three main objective, first one is of identity and demographic profile in MMS in hospital based care, needs clarity as stated above.**

**Second objective based on APEASE approach, but more details on methodology of each variable needs to be described properly.**

**Third objective specifically based on psychometric evidence only, but what method used not clearly mentioned.**

**Answer: **We appreciate the reviewer drawing our attention to a lack of clarity within the three main objectives. For **Objective 1**, in line with the updates made to the title and scope, we have revised the first objective to explicitly reflect our focus on adult inpatients within acute care hospital settings. For **Objective 2**, we have provided further detail, and have described concrete, context-specific examples for each criterion (e.g., patient willingness to contribute within bedside interviews for Acceptability) offering clarity on the structured and rigorous evaluation we will conduct of how these safety measures function within acute care environments. For **Objective 3**, we have offered more detail on plans to summarize psychometric evidence. In addition to these objectives, we have outlined an additional **fourth objective** that our review will seek to address (i.e., to identify barriers and facilitators to implementing and engaging with patient-informed safety measures in the inpatient acute care setting). We hope these changes will be satisfactory to the reviewer. We have tried to balance providing important information with offering clear, reader-friendly, and concise objectives. We believe this, coupled with additional detail added to the method section, provides the necessary information for readers.

**3. The methods section need more explanation on how the data collected as stated above and what method used to analyse need to mentioned.**

**Answer: **We appreciate the reviewer's feedback. We have expanded the Data synthesis section of the Methods to provide a more comprehensive explanation of our data collection and analysis procedures. Specifically, we have contextualized the data extracted related to the APEASE criteria, by integrating explicit operational definitions for all six dimensions of the APEASE framework in the context of patient-informed safety measurement in acute hospital settings (see **Data synthesis, Feasibility and contextual appropriateness**). Furthermore, we have clearly defined how the psychometric properties of validity, reliability, and usability will be operationalized and categorized within our synthesis framework (see **Data synthesis, Psychometric evidence**). These additions clarify both the specific types of data that will be extracted and the thematic structure that will guide our qualitative analysis. In addition to these revisions, the Data synthesis section also outlines how we will extract and synthesize data related to our newly-added fourth objective. Specifically, we describe how barriers and facilitators to implementing and engaging with patient-informed safety measures in the inpatient acute care setting will be categorized using the behavior theory framework referred to as the COM-B (Capability, Opportunity, Motivation- Behaviour) model (see **Data synthesis, Barriers and facilitators**).

**4. A short note on expected outcome may be added.**

**Answer: **The authors agree that a short note on the expected outcomes of this review would be useful to readers. We have updated the manuscript to include a dedicated "Expected Outcomes" section within the Discussion to clearly outline the explicit contributions of this review. Specifically, we have summarized the four key structural outputs of our synthesis: a comprehensive categorization of existing patient- and family-facing safety measurement tools; a multi-dimensional evidence synthesis balancing psychometric properties with the APEASE feasibility framework; a theory-driven mapping of the behavioral barriers and facilitators to patient involvement in safety data collection, using the COM-B model; and context-specific best-practice guidance for researchers and clinicians (see **Discussion, Expected outcomes**). This addition clearly highlights how the review will provide an actionable foundation for integrating the patient's voice into safety surveillance.

# Reviewer report: Reviewer response for version 1

**DOI:** 10.21956/hrbopenres.15897.r55130

**Published:** 2026-06-03

**Authors:** Leila Keshtkar

**Affiliations:** 1 University of Leicester, Leicester, England, UK

The protocol is generally well structured, methodologically rigorous, and aligned with established guidance for umbrella reviews. The review addresses an important and timely topic and demonstrates strong engagement with relevant methodological frameworks.

 The rationale for the review is generally clear and well developed. However, some statements within the introduction appear somewhat overstated. In particular, the claim that patient safety initiatives have “largely ignored” the patient voice does not fully acknowledge the growing international emphasis on patient-centred care and patient engagement in safety. The introduction would benefit from a more balanced discussion distinguishing general patient involvement from involvement specifically in safety measurement and monitoring.

 The methods are described in substantial detail and are generally sufficient to allow replication. The protocol clearly outlines the search strategy, eligibility criteria, screening procedures, quality appraisal methods, and synthesis approach. However, the temporal coverage of database searches is not clearly specified, and reporting the search timeframe would improve transparency and reproducibility. In addition, further clarification regarding the inclusion criteria for “comprehensive reviews” beyond systematic reviews would strengthen methodological transparency.

 The proposed flexible coding strategy is appropriate given the anticipated heterogeneity of approaches. However, additional detail regarding how coding disagreements, category refinement, and reviewer consistency will be managed would further improve reproducibility.

 As this is a protocol, no final datasets or synthesis outputs are yet available for evaluation. Nevertheless, the manuscript would benefit from additional detail regarding how complex findings and overlap between included reviews will be presented in accessible and interpretable formats.

 Overall, this is a strong and methodologically sound protocol with clear potential to make an important contribution to the patient safety literature.

Is the study design appropriate for the research question?

Yes

Is the rationale for, and objectives of, the study clearly described?

Yes

Are sufficient details of the methods provided to allow replication by others?

Yes

Are the datasets clearly presented in a useable and accessible format?

Partly

Reviewer Expertise:

Patient safety, communication, evidence synthesis, health services research.

I confirm that I have read this submission and believe that I have an appropriate level of expertise to confirm that it is of an acceptable scientific standard, however I have significant reservations, as outlined above.

# Response

**Published:** 2026-07-14

**Authors:** Róisín O'Malley

**Affiliations:** Discipline of General Practice, University of Galway, Galway, Co. Galway, Ireland

**The protocol is generally well structured, methodologically rigorous, and aligned with established guidance for umbrella reviews. The review addresses an important and timely topic and demonstrates strong engagement with relevant methodological frameworks.**

 Thank you for your constructive and kind review of our protocol, we believe your suggestions have strengthened the clarity and focus of our review further.

**1. The rationale for the review is generally clear and well developed. However, some statements within the introduction appear somewhat overstated. In particular, the claim that patient safety initiatives have “largely ignored” the patient voice does not fully acknowledge the growing international emphasis on patient-centred care and patient engagement in safety.** **The introduction would benefit from a more balanced discussion distinguishing general patient involvement from involvement specifically in safety measurement and monitoring.**

**Answer: **The authors thank the reviewer for this insightful comment. We have revised the introduction to provide a more nuanced, balanced background to our work. Specifically, we tempered claims around the exclusion of the patient voice and now explicitly acknowledge the growing international emphasis on patient empowerment and patient-centred care (see **Intro, paragraph 1**). Further, we have improved clarity by explicitly distinguishing general patient involvement in healthcare decision-making (e.g., treatment preferences, clinical plans, and self-management) from the specific mechanism of involving patients and their families directly in patient safety measurement (see **Intro, paragraph 2**). We believe these changes offer a much more balanced rationale for the current review.

**2. The methods are described in substantial detail and are generally sufficient to allow replication...However, the temporal coverage of database searches is not clearly specified, and reporting the search timeframe would improve transparency and reproducibility.**

**Answer: **The authors agree that explicitly outlining the search timeframe would further enhance the transparency and reproducibility of our search strategy. Within the Search strategy section of the methodology (see **Search strategy, paragraph 1**), we have updated the text to clarify that no publication year limits will be applied, explicitly stating that the searches will cover literature from database inception up to April 2026.

**3.  In addition, further clarification regarding the inclusion criteria for “comprehensive reviews” beyond systematic reviews would strengthen methodological transparency.**

**Answer: **The authors agree that the term ‘comprehensive reviews’ is somewhat unclear. In the revised manuscript, we have removed the ambiguous term “comprehensive reviews” and state that eligible reviews of any design type will be eligible for inclusion if they include an explicit methods section and demonstrate a systematic search strategy across at least two databases (see **Eligibility criteria, Study type, paragraph 1**). We hope the reviewer agrees that this is now more clearly presented.

**4.   The proposed flexible coding strategy is appropriate given the anticipated heterogeneity of approaches. However, additional detail regarding how coding disagreements, category refinement, and reviewer consistency will be managed would further improve reproducibility.**

**Answer: **The authors thank the reviewer for this suggestion. To maximize the reproducibility and rigor of our analysis, we have expanded the Data synthesis section of the methods to explicitly detail how coding consistency, category refinement, and disagreements will be managed (see **Data synthesis, paragraph 1**). We have clarified our process and now detail a formal pilot of the coding framework on an initial sample of five studies, a structured meeting procedure between the two primary extractors to align data and refine categories, and the process for resolving instances where a consensus cannot be reached (i.e., consultation with additional members of the research team).

**5.   As this is a protocol, no final datasets or synthesis outputs are yet available for evaluation. Nevertheless, the manuscript would benefit from additional detail regarding how complex findings and overlap between included reviews will be presented in accessible and interpretable formats.**

**Answer: **Within the Data synthesis section of the manuscript, we describe how we will assess and report the overlap of primary research studies across the included reviews to ensure clarity on the evidence base being considered is offered. Further, we have specified that because this review focuses on a narrative synthesis of methodology rather than a meta-analysis of quantitative data, the presentation of data will centre on clear, thematic categorisation in line with similar umbrella reviews of measurement tools (Lorente et al., 2020; Moens et al., 2026; O’Connor et al., 2021). Lastly, we have highlighted that the data extraction and synthesis will be conducted by experienced researchers with advanced backgrounds in systematic review methodologies, ensuring the analytical expertise necessary to present these complex findings in a highly accessible and interpretable format (see **Data synthesis, paragraph 8**). If the reviewer would like any further specific detail, we would be happy to add this.
